# Delivering reproductive health services through non-state providers in Pakistan: understanding the value for money of different approaches

**DOI:** 10.1186/s41256-018-0089-4

**Published:** 2018-12-04

**Authors:** Adrian Gheorghe, Rashid Uz Zaman, Molly Scott, Sophie Witter

**Affiliations:** 1 Oxford Policy Management, Level 3 Clarendon House, 52 Cornmarket St, Oxford, OX1 3HJ UK; 2 Institute for Global Health and Development, Queen Margaret University, Edinburgh, UK

**Keywords:** Reproductive health, Social franchising (SF), Social marketing (SM), Value for money, Pakistan, Non-state providers

## Abstract

**Background:**

Delivering Reproductive Health Results(DRHR) programme used social franchising (SF) and social marketing (SM) approaches to increase the supply of high quality family planning services in underserved areas of Pakistan. We assessed the costs, cost-efficiency and cost-effectiveness of DRHR to understand the value for money of these approaches.

**Methods:**

Financial and economic programme costs were calculated. Costs to individual users were captured in a pre-post survey. The cost per couple years of protection (CYP) and cost per new user were estimated as indicators of cost efficiency. For the cost-effectiveness analysis we estimated the cost per clinical outcome averted and the cost per disability-adjusted life year (DALY) averted.

**Results:**

Approximately £20 million were spent through the DRHR programme between July 2012 and September 2015 on commodities and services representing nearly four million CYPs. Based on programme data, the cumulative cost-efficiency of the entire DRHR programme was £4.8 per CYP. DRHR activities would avert one DALY at the cost of £20. Financial access indicators generally improved in programme areas, but the magnitude of progress varies across indicators.

**Conclusions:**

The SF and SM approaches adopted in DRHR appear to be cost effective relative to comparable reproductive health programmes. This paper adds to the limited evidence on the cost-effectiveness of different models of reproductive health care provision in low- and middle-income settings. Further studies are needed to nuance the understanding of the determinants of impact and value for money of SF and SM.

**Electronic supplementary material:**

The online version of this article (10.1186/s41256-018-0089-4) contains supplementary material, which is available to authorized users.

## Background

Despite evidence of progress in improving maternal health outcomes since 1990, close to 300,000 maternal deaths occur every year [1, 2]. Globally, an estimated 77% of women of reproductive age have their family planning (FP) needs met with a modern method [3], however geographic, demographic and socioeconomic disparities are significant [4].

While there is agreement that contraceptives are cost-efficient and cost-effective, a research gap persists as to the costs and impacts of demand creation strategies for contraceptives to increase service coverage [5–7]. In recognition that the mere availability of health services and products is necessary, but insufficient to improve health status, demand creation strategies aim to improve awareness and acceptability among target beneficiaries – they include, but are not limited to “*development of advocacy materials for family planning; dissemination of appropriate messages for family planning by community health workers; advocacy on family planning at the community levels to involve the formal and informal leaders; sensitization and awareness creation through community radio, radio drama, television drama, etc.; targeting of special groups including male motivation etc., in the promotion of contraceptives; training of community health/extension workers and others for promotion of family planning; and social marketing of modern contraceptives*” [8]. Social marketing (SM) and social franchising (SF) are two such demand creation strategies. SM uses approaches from commercial marketing to provide contraceptive products and services at subsidized rates; a SM organization would often be responsible for managing the implementation of marketing approaches such as branding and community mobilization through a standardized protocol. Under SF, outlets (e.g. NGOs, clinics, pharmacies) run by service providers (e.g. nurses, community health workers) deliver family planning services under contracts with an agency or franchisor providing standardized products and services under a common brand [9]. Despite several decades of implementation experience [10, 11], there is yet little published evidence on whether SM and SF are effective and cost-effective approaches to deliver FP commodities and services. For instance, a recent systematic review of five private sector models of delivering basic care, including SM and SF, confirmed that the impact and economic evidence base remains weak [12]. Another systematic review focused on SF found an equally weak evidence base [13], with at least two other quasi-experimental evaluation published since showing no overall impact of SF on FP coverage [14, 15].

Maternal, child and infant mortality outcomes are poor in Pakistan and progress towards Millennium Development Goals 4 and 5 has been slow [16, 17]. Demographic and Health Survey 2012–2013 results pointed to a 35% contraceptive prevalence rate (CPR) and a 26% rate of modern contraceptive method use [18]. Low CPRs in parts of Pakistan may be attributed to insufficient physical access to methods, health concerns, cultural or religious restrictions, or the male partner opposing contraceptives use [19]. This points to a deeper issue of low demand for contraceptives, suggesting the need for behaviour change communication interventions alongside efforts to improve the supply of reproductive health (RH) services.

Under the Lady Health Workers (LHWs) Programme, launched in 1994 and later renamed the National Family Planning and Primary Health Care Program, LHWs create awareness through door-to-door meetings and provide short-term, modern FP supplies to women who express an intention to adopt FP. Although the programme was found to be associated with increased use of a modern FP method, its evaluation found that the extent to which it reaches the most disadvantaged could be improved [20]. This is particularly relevant given the demonstrated and persistent socio-economic gap in the use of FP methods in Pakistan [21].

We evaluated the cost, cost-efficiency and cost-effectiveness of a complex FP programme in Pakistan which comprised both SF and SM approaches. The results can inform planning and budgeting decisions for a potential programme scale-up, as well as, potentially, the adoption and design of similar programmes elsewhere.

## Methods

### Setting

The UK Department for International Development (DFID) funded the Delivering Reproductive Health Results (DRHR) through Non-State Providers programme to support non-state service providers over four years (2012–2016) to expand the delivery and utilisation of high-quality reproductive health services and commodities in under-served urban and rural areas in southern Punjab, northern Sindh, Khyber Pakhtunkhwa and Federally Administered Tribal Areas (FATA). The project started with two implementing partners using different approaches to scale up access to and use of modern family planning services: Marie Stopes International (MSI), working through its local branch Marie Stopes Society (MSS), used a SF model; and Population Services International (PSI), working with its local counterpart Greenstar Social Marketing (GSM), used a SM approach.

### Description of interventions

In the SF sub-programme, MSI has used the MSS ‘*Suraj*’ model whereby a network of Suraj franchises (Suraj A and Suraj B clinics) were trained on client-centred services, counselling and side-effect management. Suraj clinics are essentially partnerships with local private health services providers located in peri-urban and rural areas at an average distance of 40–80 km from district headquarters. They are supported by reproductive health private providers (RHPPs) called ‘*Pehli Kiran’*, which are supplied with contraceptives to provide short-term and intrauterine devices (IUD) services. Pehli Kiran are service providers particularly targeting far-flung rural communities and represent more than 90% of providers in the franchised network. The project trains them in community mobilisation and information, education and communication (IEC) utilising existing materials. Behaviour change communication and marketing activities involve distributing vouchers for family planning services through a network of field worker marketing agents (FWMs) to help increase the health seeking behaviour of the poor and under-served for family planning services. FWMs conduct door-to-door visits to market the Suraj brand and services, mobilise the community, generate referrals and distribute vouchers to potential clients based on a poverty assessment. The vouchers entitle those who obtain them to have an IUD insertion for free at franchised providers. Those who do not qualify for the voucher pay the full amount (200Pakistani rupees).

In the SM sub-programme, PSI has supplied SM commodities, products and advice for RH (mainly FP), along with demand side interventions, in under-served urban and rural areas. Similar to SF, the approach addresses both the demand side and the supply side of family planning/reproductive health commodity and service provision. PSI working through their affiliate, GSM, use a total market approach which includes commercial for-profit products, social marketed subsidised products and products that are free for the poorest. To empower women and girls to make healthy reproductive choices, GSM and its implementing partners reach young women, men and key influencers like health care providers, husbands and mothers in law, with evidence-informed messages promoted through interpersonal communication. In Year 3, GSM also relaunched and strengthened its toll-free helpline, which provides family planning and sexual health and reproductive health information, counselling and referrals.

### Design

We evaluated the cost, cost-efficiency and cost-effectiveness of the DRHR programme and its components (SM and SF). For the cost analysis, both programme costs and user costs were calculated. Programme costs refer to the cost of activities undertaken by the implementing partners to provide services and commodities. User costs refer to costs that individuals are subject to when accessing products and services offered as part of the programme. Monetary costs include out-of-pocket (OOP) payments to cover the sale prices of commodities/services, professional fees, transport to the service provider and, if applicable, accommodation. Non-monetary costs include the value of productive time lost by the user and anyone accompanying them to the service provider, as well as social sanctions stemming from cultural perceptions of services and others. The analysis included only monetary costs.

The cost per couple years of protection (CYP) and cost per new user were estimated as indicators of cost-efficiency. Three cost-effectiveness indicators were estimated: cost per maternal death averted; cost per unsafe abortion averted; and cost per unintended pregnancy averted.

### Data sources

To estimate programme costs, quarterly invoices and expenditure reports sent to DFID by MSI and PSI were obtained. We requested additional information from each implementing partner on: staff costs; volumes and prices of commodities purchased and disbursed; the internal charts of accounts; and cost recovery arrangements. User cost data were collected using a pre-post survey whose methodology and findings were reported elsewhere [22]. Briefly, 7888 statistically representative households were surveyed in 400 clusters at baseline (mid-2013) and 6336 households were successfully followed up at endline (late 2015). Costs and outcomes were compared between married women of reproductive age (MWRAs) in two DRHR evaluation groups (a ‘PSI only’ group and a ‘combined MSI and PSI’ group) and those who were not exposed to either PSI or MSI activities (control). The sampling strategy was based on randomly selecting evaluation clusters from one of the three groups, at the sub-district (tehsil) level. The evaluation used a combination of propensity score matching (PSM) and differences in differences methods to estimate the changes in costs and outcomes (access, utilisation, equity and quality of care) that can be attributed to the programmes. The analysis of user costs was informed by the panel dataset of women (*n* = 5514) who participated in both the baseline and endline surveys.

For cost-efficiency indicators, the cost term in ‘cost per CYP’ and ‘cost per new user’ was informed by total programme costs. CYP estimates were sourced from reports of the implementing partners.

### Analysis

Both financial and economic costs were calculated [23]. The analysis of financial costs took a top-down approach in which the programme budget was disaggregated into cost categories. All programme costs are expressed in British pounds (GBP, 2015 value). Given that implementing partners invoiced programme expenditures in GBP, expenditures incurred in 2012–2014 were first converted to Pakistani rupees (PKR), inflated to their 2015 values using Pakistan’s annual inflation rate for 2012–2014, [24] and then converted back into GBP using the average exchange rate for the last quarter of the evaluation period (July – September 2015) [25].

Economic costs were estimated as the sum of financial costs with annuitisation, the value of commodity subsidies and programme cost recovery. Financial costs with annuitisation were calculated by subtracting the value of fixed assets from financial costs, then calculating and adding back capital depreciation for each programme year. Capital depreciation was calculated using the straight line method based on the useful life in the asset register or assuming a useful life of five years (when useful life was not recorded in the asset register), and a salvage value of 10% of the acquisition price [26]. The value of commodity subsidies was estimated by multiplying the number of commodity units acquired at subsidized price by the difference between the acquisition price (assumed zero if donated) and corresponding market prices. Cost recovery (income that implementation partners make as a result of programme operations) was incorporated as a cost incurred at the moment when it was collected (subtracted from financial cost); in the absence of any indication to suggest otherwise, it was assumed that these funds were not reinvested in the programme and did not lead to additional outputs.

Two types of ‘cost per new user’ were estimated: one based on new users reported by the implementers through their field activities (PSI/GSM recorded new users from Year 2 onwards); and another calculated based on self-reported FP method use in the survey data. Given the distinctions between three types of users (users of FP methods at baseline, but not at endline; constant users or non-users at both baseline and endline; and users of FP methods at endline only), the group ‘users of FP methods at endline only’ was used to estimate total new users for the purpose of this analysis i.e. women who reported not using a modern family planning method at the baseline survey, but did report using a method at endline, and were not pregnant at either baseline or endline. We extrapolated ‘new users’ as defined above using survey sampling weights to the population from which respondents were sampled, thus estimating total new users for each evaluation group.

Cost-effectiveness indicators were estimated by dividing total programme costs by the cumulative clinical events averted as reported by each implementing partner. Additionally, disability-adjusted life years (DALYs) averted were estimated using the MSI Impact 2 calculator [27] based on commodity data provided by the implementing partners. An incremental analysis was also performed for SM and SF against each other by calculating an incremental cost-effectiveness ratio through dividing the difference in costs by the difference in outcomes averted between them. The rationale for the incremental analysis is that cost-effectiveness analysis can appropriately inform decision-making only if the intervention of interest is compared against the best available alternative. As such, a decision-maker faced with choosing either SM or SF as a model to deliver FP services, if such a decision is viable, will be interested not only in how they compare individually against ‘doing nothing’ but also against each other.

No time adjustment was applied to measures of health benefit in the cost-efficiency and cost-effectiveness analyses in the understanding that these benefits occur in the same period (e.g. year of programme) as that in which they are reported.

## Results

### Programme costs

The DRHR programme spent £19,389,941 (GBP 2015 value) between July 2012 and September 2015 on SM and SF sub-programmes (Table 1). Two thirds of DRHR funds (65%) were spent on reimbursables and the remaining 35% on staff salaries. Compared to the financial costs, economic costs were higher by approximately £885,000 (8.5% of financial costs) in the SF sub-programme and lower by approximately £1,665,000 (15% of financial costs) in the SM sub-programme. Economic costs in the SM sub-programme are underestimated considering that the value of subsidized commodities could not be calculated due to insufficient data.

**Table 1:** Economic and financial costs of the DRHR programme

	SM	SF	Total
Financial costs (GBP)			
Fees	1,084,009	6,052,910	7,136,919
Reimbursables	8,308,875	5,168,704	13,477,579
Total	9,392,884	11,221,614	20,614,499
Economic costs (GBP)			
Equipment cost	132,324	1,279,427	1,411,751
Capital depreciation	114,113	350,409	464,522
Commodity subsidies	NA	46,469	46,469
Cost recovery	458,602	2726	461,328
Total	9,833,275	10,341,792	20,175,067

MSI and PSI expenditure data and OPM calculations

NA – data not available. Costs cover the entire duration of the programme up to when this analysis was conducted (Q1-Q13) and are calculated as: Total financial cost (2015 value) – equipment cost + capital depreciation + commodity subsidies + cost recovery

### User costs

The proportion of clients who reportedly incurred transport costs to reach the nearest family planning method provider remained constant in all arms of the impact evaluation (Table 2). Respondents in the control arm spent on average 65 PKR less (*p*-value 0.05) on transport at endline than at baseline, while in the SM and SM + SF arm transport cost differences were of similar magnitude and were not statistically significant.

**Table 2:** User costs for transport and FP methods

	Control			SM only			SM and SF		
	Baseline	Endline	Difference	Baseline	Endline	Difference	Baseline	Endline	Difference
Transport cost to nearest family planning method provider									
Of those visiting RH provider, % that spent any money on transport	26.7	21.6	−0.05	52.5	55.4	0.03	28.3	31.4	0.03
Mean spend on transport to each nearest RH provider (PKR)	140.4	75.8	−64.7**	96.0	159.6	63.6	127.7	70.6	−57.1
Cost to obtain family planning method at provider									
% MWRAs spending any money to obtain contraceptive method last time	50.8	24.6	−26.2*	53.2	69.5	16.3**	43.8	34.5	− 9.3
Mean spend on obtaining contraceptive method last time (PKR)	68.9	579.0	510.1	254.1	121.3	− 132.8**	146.9	152.9	6.0

OPM analysis of MSI/PSI baseline and endline surveys

Asterisks indicate that the difference between the baseline and endline value is statistically significant

*= significant at 10% level

**= significant at 5% level

***= significant at 1% level

In the control arm the proportion of respondents who had to pay anything to obtain contraceptive method decreased from 51 to 25% (p 0.06) and the mean spend on contraceptive methods increased by 510 PKR, though this increase was not statistically significant (Table 2). In the SM-only arm, more respondents had to pay to obtain contraceptive methods, however the average amount spent decreased by 133 PKR (*p*-value 0.03). There were no significant changes from baseline in the SM + SF arm. The average spending differences require a cautious interpretation because of the limited number of responses in each arm (*n* < 100) as this question was asked only to survey respondents who were: current users of FP, had obtained FP in the past 3 months, and had to pay something for their FP.

### Cost-efficiency

In total 3,987,517 CYPs were delivered across the two sub-programmes, leading to a cumulative cost-efficiency of £4.76 per CYP (GBP 2015 value). The average cost per CYP delivered was £5.69 in the SF sub-programme and £4.10 in the SM sub-programme (Table 3). Estimated economic costs per CYP were lower than financial costs for the SF programme (£5.21) and higher in the SM programme (£4.37).

**Table 3:** Cost per CYP in DRHR

Indicator	SM	SF	Total
CYPs achieved	2,151,994	1,835,523	3,987,517
Financial costs (GBP)	8,833,446	10,436,488	19,269,934
Financial costs per CYP	4.10	5.69	4.83
Economic costs (GBP)	9,412,820	9,556,665	18,969,485
Economic costs per CYP	4.37	5.21	4.76

At the time of the analysis MSI reported actual CYPs only for the period July 2012 – June 2015 (Q1-Q12, excluding Q13). For consistency, in this analysis costs and CYPs incurred during quarters 1–12 were considered for both SM and SF. Financial and economic costs for both SF and SM are lower than reported in Table 1, the differences representing costs incurred during Q13

MSI estimated that the SF sub-programme attracted 462,542 new users from inception to June 2015. When considering the £10,436,488 (2015 value) expenditure during the same period, this leads to an estimated £22.6 per new user. PSI/GSM reported 162,832 new users for Year 2 and Year 3. Considering the £6,876,494 (2015 value) sub-programme expenditure incurred during the same period, this leads to an estimated £42.2 per new user.

**Fig. 1:**
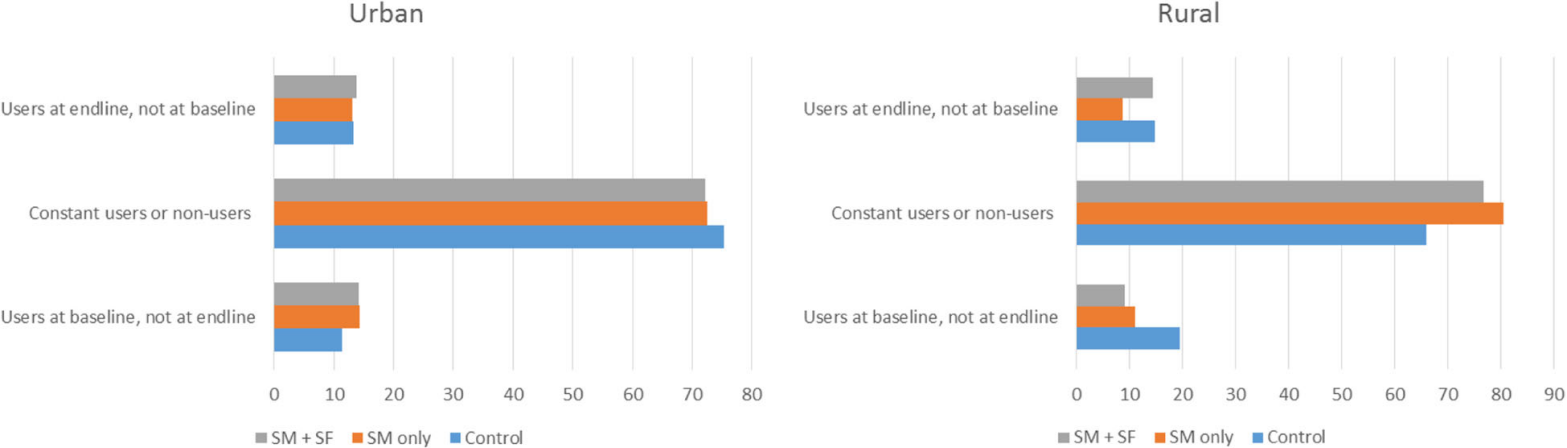
Estimated users at baseline and endline, by residence (%). Survey data and OPM calculations

Approximately 14% of survey respondents in MSI and PSI areas appear to be new users, more than in PSI only areas (9.1%) and closely comparable to control areas (Fig. 1). There is evidence of discontinuation, reflected in self-reported users at baseline who were not family planning methods users at endline: 10% in MSI and PSI areas, less than in control (18%) and comparable to PSI only areas (11%). By extrapolating survey data based on survey sampling weights we estimated 216,791 new users in SM + SF areas, pointing to a cost of £43.6 per new user for the SF sub-programme.

### Cost-effectiveness

We estimated a cost of £20 per DALY averted and £21 per unintended pregnancy averted for the DRHR programme (Table 4). For the SF sub-programme we estimated a cost of £22 per DALY averted and £11,258 per maternal death averted. For the SM sub-programme, the cost per unintended pregnancy averted is somewhat higher than for SF (£23 compared to £16), while the cost per abortion averted is somewhat lower (£104 compared to £148). With the exception of unsafe abortions, the SF sub-programme was more costly and also more effective than the SM sub-programme. When comparing SM to SF in an incremental analysis, SF would avert one unintended pregnancy for an additional expense of £3, avert one maternal death for an additional £3324 and avert one DALY for an additional £24.

**Table 4:** Cost-effectiveness indicators for DRHR

Indicator	SF	SM	Total	Incremental analysis (SF-SM)
Unintended pregnancies	648,629	300,763	949,392	347,866
Maternal deaths	927	613	1540	314
Unsafe abortions	70,673	70,860	141,533	−187
DALY averted	517,423	473,737	991,160	43,686
Financial costs (GBP)	10,436,488	9,392,884	19,829,372	1,043,604
Cost per unintended pregnancy averted	16	23	21	3
Cost per maternal death averted	11,258	12,145	12,876	3324
Cost per unsafe abortion averted	148	104	140	SF is dominated
Cost per DALY averted	22	20	20	24

MSI and PSI data and OPM calculations

Cost and outcomes data on unintended pregnancies averted, maternal deaths averted and unsafe abortions averted are as supplied by MSI (up to June 2015). Cost per unintended pregnancy averted, maternal death averted and unsafe abortion averted for MSI are informed by programme costs up to June 2015 (£10,438,488). DALYs averted were calculated using the MSI Impact 2 model based on the product mix data supplied by MSI and PSI. All DALYs averted until 2019 (the longest-acting family planning method delivered in DRHR, other than sterilisation, was assumed to provide protection for 5 years) were aggregated without discounting

## Discussion

### Summary of findings

Approximately £20 million were spent through the DRHR programme between July 2012 and September 2015 on commodities and services representing nearly four million CYPs. Based on programme data, the cumulative cost-efficiency of the entire DRHR programme was £4.8 per CYP (2015 value). Similarly, DRHR activities would avert one DALY at the cost of £20. Financial access indicators generally improved in programme areas, but the magnitude of progress varies across indicators. Improvements in control areas have been comparable to – if not better than – improvements in programme areas [22, 28]. We present key findings of the impact assessment in Additional file 1: Appendix 1.

### Interpretation of findings

Our findings need to be viewed in reference to the broader family planning context in Pakistan. While Demographic and Health Survey (DHS) data indicate reductions in unmet need for family planning over time (from 31% in 1990–91 to 17% in 2017–18), the use of modern contraceptive methods and proportion of women with demand satisfied with modern contraceptive methods have remained largely stationary in Pakistan over the past five years at 25 and 49%, respectively [29]. The cultural determinants of access to family planning services and products are complex in Pakistan – while knowledge of at least some contraceptive methods may be high, there are multiple barriers to contraceptive use which include but are not limited to: religion (e.g. religious imperative to have as many children as possible), fear of side-effects (e.g. bleeding after contraceptive injection), social stigma (e.g. disapproval in the community), family stigma (e.g. pressure from husband or in-laws), limited female mobility (e.g. women not allowed to travel alone) and others [19]. The decision to use contraceptives is hardly an individual one, as the views of a woman’s husband and in-laws may often prove decisive.

Assessing whether DRHR offered good value for money depends on the availability of acceptable benchmarks. In the absence of universal benchmarks for the cost per CYP and the cost per new user, we compared our findings with those of similar programmes. We identified in the University of California at San Francisco (UCSF) Clinical Social Franchising Compendium 2014 [30] seven SF programmes (in Democratic Republic of Congo, Guatemala, Haiti, Madagascar, Malawi, Senegal and Sierra Leone) for which we could calculate the cost per CYP, which ranged from £3.5 (Senegal) to £92.5 (Haiti), with five of seven estimates below £10 per CYP (Additional file 1: Appendix 2). An evaluation of an injectable contraceptive program combining community-based distribution and SM in Ethiopia found an average programmatic cost of $17 per CYP (approximately £13) and a direct cost service cost of $2 (approximately £1.5) [31]. Two annual reviews of DFID-funded RH projects reported a cumulative cost of £14.5 per CYP and £13 per additional user (Zambia [32]); and £6.4 per CYP (nine countries in sub-Saharan Africa and five countries in southern Asia [33]). Finally, estimates for Pakistan suggest the public sector delivers FP services at an average cost of $17 (approximately £13) per CYP [34]; and a modelling study looking at social marketing alone suggested an average incremental cost of $4.3 (approximately £3.2) per CYP [35]. While the findings of these studies are difficult to compare directly because of differences due to setting (e.g. country, urban/rural mix of providers and beneficiaries), programme design (e.g. scale and mix of contraceptive interventions) and methodology (e.g. impact and cost estimation), their results are consistent in suggesting a range of £3 to £15 for the total cost per CYP. Given that DRHR and its sub-programmes averaged below £6 per CYP, this suggests they are likely to be cost-efficient. Some caution is needed, however, because cost per new user estimates are scarce and difficult to estimate robustly, therefore cost-efficiency is largely informed by cost per CYP estimates.

External benchmarks are available for the cost per DALY averted. Thresholds informed by countries’ gross domestic product (GDP) per capita were long used in global health [36, 37], informed by the work of the Commission for Macroeconomics and Health [38], before the World Health Organization recommended country-specific thresholds for decision-making [39]. The International Decision Support Initiative (iDSI) updated these thresholds based on the likely marginal productivity of health systems and suggested a cost-effectiveness threshold range for Pakistan of $87–669 (approximately £58–448) per DALY averted [40]. Furthermore, for the seven programmes in the UCSF Social Franchising Compendium we calculated a cost per DALY averted ranging from £2.5 (Sierra Leone) to £133.7 (Haiti), with six of seven values below £50 per DALY averted. The DRHR, SF and SM cost per DALY averted estimates compare favourably against these values, suggesting they can be considered cost-effective.

It is difficult to say whether SF or SM offered better value in DRHR. First, the indicators send a mixed message. In terms of cost-efficiency, the SF sub-programme had a slightly higher cost per CYP (£5.69 vs £4.04) and a lower cost per new user (£22 vs £42) than the SM sub-programme. In terms of cost-effectiveness, SF and SM achieved comparable performance for the cost per DALYs averted (£22 for SF and £20 for SM), maternal death averted (£11,258 vs £12,145) and unintended pregnancy averted (£16 vs £23); the only exception was the cost per unsafe abortion averted, which was higher in the SF programme (£148 vs £104).

Secondly, caution is warranted when comparing SF and SM directly because they take different approaches to delivering impact, with implications for the structure of expenditures and outcomes. For example, start-up costs are higher for the SF sub-programme, given the need to attract and train new franchisees, while the SM programme relies to a larger extent on an existing network of providers. Furthermore, they take different approaches to delivering CYPs: the SF sub-programme relied primarily on long-term methods (97% of CYPs), while in the SM approach the product mix was more diverse i.e. condoms (49% CYPs), IUDs (30%) and contraceptive pills (11%). Differences between estimates of economic and financial costs illustrate the consequences of such differences on estimating value for money. Economic costs were lower than financial costs for the SF sub-programme, a consequence of the capital-intensive nature of the delivery model; and higher for the SM sub-programme, given programme revenues. The latter would have been even higher if sufficient data had been available to incorporate the value of commodity subsidies.

There is little evidence to suggest that DRHR activities contributed to narrowing disparities in access to FP methods in Pakistan. First, geographical and financial access improved for both urban/rural and poor/non-poor respondents [22]. Secondly, the rural and poor also started from an inferior baseline and the fact that relative improvements are comparable to those observed among the better off signals that more could have been done and remains to be done to achieve convergence. Furthermore, the absolute improvements in financial and geographical access indicators were generally small.

### Limitations

Most limitations in the analysis stem from insufficient data at the appropriate level of disaggregation. Programme costs could not be disaggregated by activity and locality (rural/urban). We attempted to conduct a top-down activity-based costing exercise, but we could allocate less than 40% of programme expenditure to specific types of activities (for both MSI and PSI), therefore we did not include this component in the analysis. Difficulties of detailed time sheets proved to be a major obstacle.

Economic costs are likely to be underestimates. First, commodity subsidy data were incomplete. Secondly, they did not incorporate household-level costs, e.g. out-of-pocket payments for products/services and transport costs. We decided not to incorporate them in the analysis given: i) the limited number of respondents who reportedly had to pay for services; ii) the low resulting cost share relative to the total programme expenditure; and iii) the less than conclusive results in regard to cost reduction. A full incorporation of the economic costs would likely make the programme and its components appear to offer less value for money than currently estimated.

CYP and commodity data were sourced from the implementers’ reports and it was beyond the scope of this analysis to verify the robustness of these data. We assumed these values as correct and comparable between MSI and PSI. However, comparability may have been affected by slight differences in methodologies and technique, e.g. applying the United States Agency for International Development (USAID) conversion factors to commodities or using the MSI Impact 2 calculator.

Finally, caution must be applied towards the ‘new users’ estimates because they are sensitive to the definition of ‘new users’. It is difficult to ascertain the extent to which MSI’s and PSI’s new users tracking mechanisms cover the entire spectrum of new users and generate results that are fully comparable with survey-informed estimates. A fundamental difference between the two types of estimates is that the survey allows for a counterfactual while the implementers’ user tracking mechanisms do not. Furthermore, the difficulties in conducting the activity-based costing prevented us from using marketing and promotion costs in the cost per new user formula, which would have led to a better estimation. Assuming the new user estimates are correct, our current results most likely overestimate the cost per new user.

### Implications for policy

Our findings suggest that using SM and SF approaches to increase FP coverage can represent good value for money in Pakistan and similar contexts. A previous quasi-experimental evaluation examining the impact of MSI’s SF approach in the country also found a positive impact on utilization [41]. Furthermore, our findings suggest that using SM and SF in combination, particularly in rural areas, may be associated with less discontinuation and more uptake of new contraceptive users compared with SM in isolation. Nevertheless, when considering the scale-up of such initiatives policy makers need to be careful not to overestimate their effectiveness or their reach among the most disadvantaged. A recent evaluation of maternal healthcare franchises in India and Uganda found a limited ability of social franchises to reach the poorest areas [42]. As such, a more complex and context-specific set of incentives (e.g. area- or income-based schedule of subsidies) and complementarity with supply-side initiatives should be considered to maximize the potential of SM and SF approaches.

### Further research

The reporting and evaluation of future similar programmes can benefit from focusing on several aspects. One is more effort towards detailed and harmonised accounting reporting standards across programme partners. Specifically, this would involve collecting expenditure data as close to the end user as possible, as well as introducing activity-oriented budget lines for both staff and capital, allowing implementers and funders alike to react swiftly to programme developments. Another is including a qualitative research component to facilitate a better understanding of which implementation aspects of the SM and SF approaches drive impact and value for money. Possible areas of exploration include: for beneficiaries – the extent to which programme activities address key context-specific barriers to contraceptive use (e.g. cultural factors) and reasons for discontinuation; and the interactions of the programme with other FP and health-related programmes. For implementers, it would be important to establish the extent to which the processes for selecting and overseeing franchisees (outlets and clinics) contribute to programme objectives.

We could only find limited research on the value of SM approaches in delivering reproductive health services and commodities – by contrast, more research has been done for SF. It remains difficult to assess the relative merits of SF and SM. More research is necessary to document the implementation, cost and impact of SM programmes globally.

## Conclusions

Our findings add to the scarce literature on the value for money of market-based approaches to deliver FP results in low- and middle-income settings. Results suggest that SM and SF generally provided good value for money in Pakistan as part of the DRHR programme when compared with other programmes. When considering the replication or scale-up of such interventions, in order to maximize impact policy makers need to consider carefully how the interventions will reach those who are most in need, what specific schedule of subsidies or other incentives is most appropriate for each population sub-group or geographical area, and how demand creation activities will complement ongoing supply-side initiatives. Subsequent, more comprehensive evaluations are needed to nuance the understanding of the determinants of impact and value for money in approaches to improving reproductive health outcomes.

## Additional file

Additional file 1: **Appendix 1.** Selected findings from the DRHR impact evaluation. **Appendix 2.** Cost-efficiency and cost-effectiveness estimates for reproductive health social franchising programmes in the UCSF Clinical Social Franchising Compendium. (DOCX 100 kb)

## Abbreviations

CPR: Contraceptive Prevalence Rate
CYP: Couple Years of Protection
DALY: Disability-Adjusted Life Year
DFID: UK Department for International Development
DRHR: Delivering Reproductive Health Results
FATA: Federally Administered Tribal Areas
FHM: Field Worker Marketing Agent
FP: Family Planning
GDP: Gross Domestic Product
GSM: Greenstar Social Marketing
iDSI: International Decision Support Initiative
IEC: Information, education and communication
IUD: Intrauterine device
LHW: Lady Health Worker
MSI: Marie Stopes International
MSS: Marie Stopes Society
MWRA: Married women of reproductive age
OOP: Out-of-pocket
PKR: Pakistani Rupee
PSI: Population Services International
PSM: Propensity Score Matching
RH: Reproductive Health
RHPP: Reproductive Health private providers
SF: Social Franchising
SM: Social Marketing
UCSF: University of California at San Francisco
USAID: United States Agency for International Development
