# Supplementary material for: Delivering reproductive health services through non-state providers in Pakistan: understanding the value for money of different approaches
Source: Glob Health Res Policy. 2018 Dec 4;3:33. doi: 10.1186/s41256-018-0089-4 (PMC6278166; doi:10.1186/s41256-018-0089-4)
Supplement: Supplementary file 1 — Appendix 1. Selected findings from the DRHR impact evaluation. Appendix 2. Cost-efficiency and cost-effectiveness estimates for reproductive health social franchising programmes in the UCSF Clinical Social Franchising Compendium. (DOCX 100 kb) [file 41256_2018_89_MOESM1_ESM.docx]

**Additional file 1**

**Appendix 1. Selected findings from the DRHR impact evaluation**

The impact estimates, which used propensity score matching to remove hidden selection bias, found no significant impact for the two intervention groups, compared to the control, for increased access to RH (reduced time and payments to reach providers) (Table 1) and contraceptive uptake (Table 2).

There was some evidence of increased exposure to communication materials in both intervention groups (Table 3). Impact estimates also found a significant increase in the mean number of modern methods named by MWRAs in the PSI+MSI group, compared to the control (Table 4).

Table 1: Impact estimates for contraceptive access

|  | PSI-only vs control | | | | PSI+MSI vs control | | | |
| --- | --- | --- | --- | --- | --- | --- | --- | --- |
|  | **Average treatment effect** | **Standard error** | **T statistic** | **N** | **Average treatment effect** | **Standard error** | **T statistic** | **N** |
| Mean time to reach nearest RH provider (in minutes) | 1.12 | 1.427 | 0.782 | 1610 | -2.22 | 1.31 | -1.692 | 1670 |
| Of those visiting RH provider, % that spend any money on transport | -0.02 | -0.481 | -0.481 | 1600 | -0.01 | 0.031 | -0.424 | 1649 |

Table 2: Impact estimates for contraceptive utilisation

|  | PSI-only vs control | | | | PSI+MSI vs control | | | |
| --- | --- | --- | --- | --- | --- | --- | --- | --- |
|  | **Average treatment effect** | **Standard error** | **T statistic** | **N** | **Average treatment effect** | **Standard**  **error** | **T statistic** | **N** |
| % MWRAs ever used contraceptive method | -0.02 | 0.024 | -1 | 2532 | -0.03 | 0.022 | -1.133 | 2524 |
| % MWRAs ever used modern method | -0.01 | 0.022 | -0.663 | 2534 | 0 | 0.021 | -0.119 | 2522 |
| % MWRAs ever used traditional method | -0.04 | 0.029 | -1.285 | 2545 | -0.05 | 0.028 | -1.72 | 2528 |
| % MWRAs currently using contraceptives^[[1]](#footnote-1)^ | 0.02 | 0.026 | 0.794 | 2354 | -0.01 | 0.025 | -0.306 | 2341 |
| % MWRAs currently using modern methods of contraception | 0.02 | 0.022 | 0.882 | 2574 | -0.01 | 0.021 | -0.52 | 2554 |
| % MWRAs currently using traditional methods of contraception | 0.02 | 0.018 | 0.887 | 2574 | 0.03 | 0.017 | 1.619 | 2554 |

* = | t-statistic | > 1.96, ** = | t-statistic | > 2.58

Table 3: Impact estimates for awareness of communication campaigns on FP

|  | PSI-only vs control | | | PSI+MSI vs control | | |
| --- | --- | --- | --- | --- | --- | --- |
|  | **Average treatment effect** | **Standard error** | **T statistic** | **Average treatment effect** | **Standard**  **error** | **T statistic** |
| % MWRAs who have seen any communication materials on contraception in the last month | 0.09** | 0.028 | 3.166 | 0.13** | 0.027 | 4.883 |
| % MWRAs who have seen any communication campaigns on contraception in the past month | -0.02 | 0.013 | -1.844 | -0.01 | 0.012 | -0.95 |
| % MWRAs who have seen anything on contraception in the media in the past month | 0 | 0.018 | 0.25 | 0.02 | 0.017 | 0.989 |

* = | t-statistic | > 1.96, ** = | t-statistic | > 2.58

Table 4: Impact estimates for contraception awareness

|  | PSI-only vs control | | | PSI+MSI vs control | | |
| --- | --- | --- | --- | --- | --- | --- |
|  | **Average treatment effect** | **Standard**  **error** | **T statistic** | **Average treatment effect** | **Standard**  **error** | **T statistic** |
| Mean number of methods that MWRAs can name | -0.17 | 0.122 | -1.381 | 0.01 | 0.114 | 0.051 |
| Mean number of modern methods that MWRAs can name | -0.01 | 0.095 | -0.106 | 0.23* | 0.088 | 2.562 |
| % MWRAs who cannot say which method is most effective | -0.01 | 0.028 | -0.316 | 0.01 | 0.026 | 0.504 |

The matching algorithm selected performed very well in terms of achieving statistical balance between treatment and control groups after matching, according to their overall characteristics. An illustration of the improvement in standardised bias associated with each variable after matching is given in Figure 1 below. Prior to matching, the figure shows that some variables were associated with a high degree of bias; however, after matching is performed the balance across these variables is shown to be markedly reduced.

Figure 1: Covariate balance for the main model before and after matching


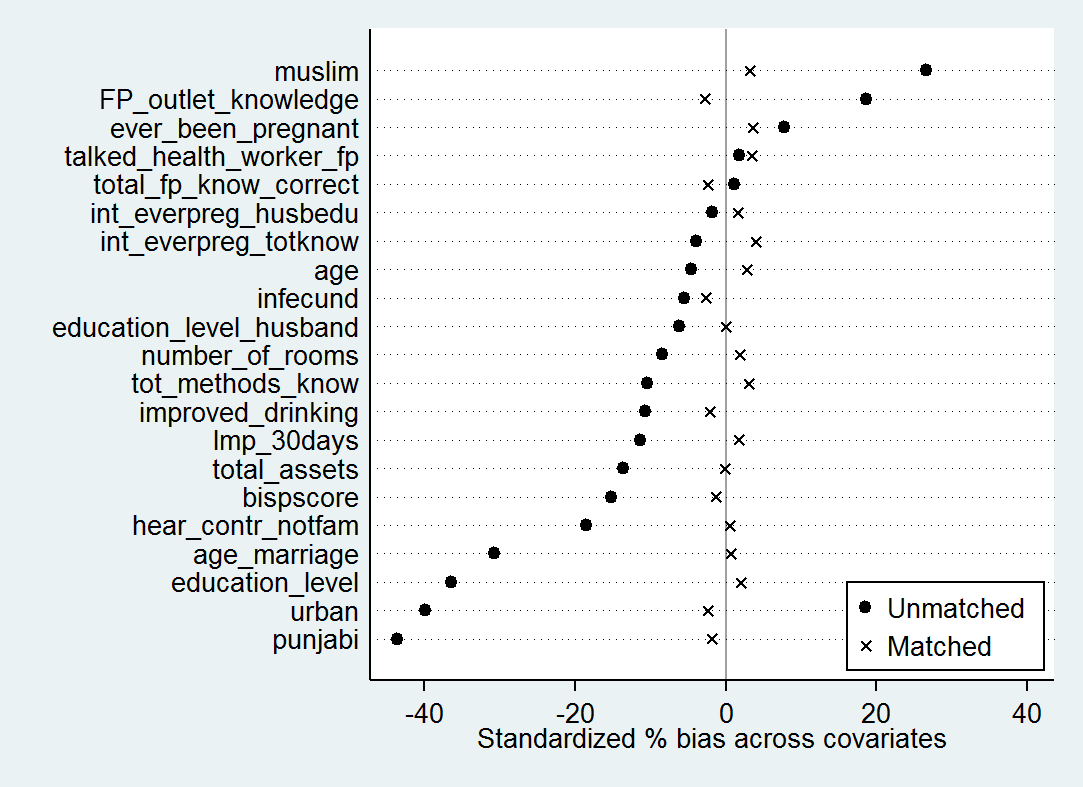


Note: This figure shows standardised bias associated with all the covariates included in the PSM model. It corresponds to the analysis performed to analyse current contraceptive use, in the PSI+MSI group vs the control group. The matching algorithm used is kernel matching (Gaussian), with trimming set at 5 and a bandwidth parameter of 0.03.

**Appendix 2. Cost-efficiency and cost-effectiveness estimates for reproductive health social franchising programmes in the** **UCSF Clinical Social Franchising Compendium**

| Country | Franchise name | Agency | Programme costs ($2013) | DALYs averted | Cost ($) per DALY averted | Cost ($) per CYP | Cost (£) per DALY averted | Cost (£) per CYP |
| --- | --- | --- | --- | --- | --- | --- | --- | --- |
| Democratic Republic of Congo | Reseau Confiance | PSI | 2,000,000 | 190,827 | 10.48 | 12.04 | 6.70 | 7.69 |
| Guatemala | Red Segura | PSI | 1,099,022 | 17,031 | 64.53 | 16.52 | 41.24 | 10.56 |
| Haiti | Plis Kontwol | PSI | 288,034 | 1,398 | 206.03 | 144.67 | 131.66 | 92.44 |
| Madagascar | BlueStar and CSB Star | MSI | 560,304 | 59,342 | 9.44 | 6.14 | 6.03 | 3.92 |
| Malawi | Tunza Family Health Network | PSI | 441,173 | 9,145 | 48.24 | 44.66 | 30.83 | 28.54 |
| Senegal | BlueStar | MSI | 77,000 | 11,973 | 6.43 | 5.48 | 4.11 | 3.50 |
| Sierra Leone | BlueStar Healthcare Network | MSI | 169,942 | 44,207 | 3.84 | 5.31 | 2.46 | 3.39 |

Source: UCSF Social Franchising Compendium 2014. Notes: Cost per DALY averted and cost per CYP are OPM calculations based on Compendium data. Conversion from USD to GBP was informed by the average USD/GBP exchange rate between 01 January 2013 and 31 December 2013 (1 USD = 0.639 GBP), available from [www.oanda.com](http://www.oanda.com).

1. The results for this indicator may be interpreted as being able to reveal whether or not the interventions have led to an increase in new users of contraception in the treatment groups, where a new user is defined as a woman who was not practising contraception at the time of the baseline survey but is practising it by the time of the endline survey. The reason why the findings on contraceptive use may be interpreted in this way is due to the fact that our analysis methodology includes a differences in differences element. This means that we are comparing a difference between baseline and endline in each indicator for women in one of the treatment groups with the corresponding difference or women in the control group. If the impact on current use of contraception is found to be positive, the interpretation would be that there are a greater number of new users in the treatment group than in the control group at the time of the follow-up survey, which is attributable to the interventions. [↑](#footnote-ref-1)
